# Modulation of the ∽20-Hz motor-cortex rhythm to passive movement and tactile stimulation

**DOI:** 10.1002/brb3.328

**Published:** 2015-03-31

**Authors:** Eeva Parkkonen, Kristina Laaksonen, Harri Piitulainen, Lauri Parkkonen, Nina Forss

**Affiliations:** 1 Department of Neuroscience and Biomedical Engineering, Aalto University School of ScienceEspoo, Finland; 2 Aalto NeuroImaging, MEG-Core, Aalto University School of ScienceEspoo, Finland; 3 Clinical Neurosciences, Neurology, University of Helsinki and Helsinki University HospitalFinland

**Keywords:** Beta rebound, beta rhythm, magnetoencephalography, motor-cortex excitability, proprioception, sensorimotor integration

## Abstract

**Background:**

Integration of afferent somatosensory input with motor-cortex output is essential for accurate movements. Prior studies have shown that tactile input modulates motor-cortex excitability, which is reflected in the reactivity of the ∽20-Hz motor-cortex rhythm. ∽20-Hz rebound is connected to inhibition or deactivation of motor cortex whereas suppression has been associated with increased motor cortex activity. Although tactile sense carries important information for controlling voluntary actions, proprioception likely provides the most essential feedback for motor control.

**Methods:**

To clarify how passive movement modulates motor-cortex excitability, we studied with magnetoencephalography (MEG) the amplitudes and peak latencies of suppression and rebound of the ∽20-Hz rhythm elicited by tactile stimulation and passive movement of right and left index fingers in 22 healthy volunteers.

**Results:**

Passive movement elicited a stronger and more robust ∽20-Hz rebound than tactile stimulation. In contrast, the suppression amplitudes did not differ between the two stimulus types.

**Conclusion:**

Our findings suggest that suppression and rebound represent activity of two functionally distinct neuronal populations. The ∽20-Hz rebound to passive movement could be a suitable tool to study the functional state of the motor cortex both in healthy subjects and in patients with motor disorders.

## Introduction

Tactile and proprioceptive input coordinates and recalibrates motor cortex activity by regulating its excitability (Cassim et al. 2000, 2001; Gaetz and Cheyne 2006). Several studies have shown that ∽20-Hz oscillatory brain activity is initially decreased (suppression; event-related desynchronization, ERD) and subsequently increased (rebound; event-related synchronization, ERS) in response to tactile stimulation (Pfurtscheller 1981; Salmelin and Hari 1994; Hari et al. 1997; Salenius et al. 1997; Neuper and Pfurtscheller 2001). The suppression is suggested to reflect an activated state of the motor cortex, whereas the rebound has been associated to a deactivated or inhibited state of the motor cortex (Pfurtscheller et al. 1996, 1997; Cassim et al. 2000; Neuper and Pfurtscheller 2001; Takemi et al. 2013).

In studies with cats and monkeys, the primary motor cortex (MI) has been shown to receive proprioceptive input via direct thalamocortical connections (Asanuma et al. 1979; Friedman and Jones 1981), via primary somatosensory cortex (SI; areas 3a and 2) and via secondary somatosensory cortex (SII; Jones and Wise 1977; Jones et al. 1978; Jones 1983). Single-cell recordings in humans have demonstrated that proprioceptive input activates MI more than tactile input (Goldring and Ratcheson 1972). As proprioception specifically signals the internal state of the locomotor system, it is likely that it modulates motor-cortex excitability more than tactile input. Prior EEG studies have shown modulation of the ∽20-Hz motor cortex rhythm to passive movement (Cassim et al. 2001; Alegre et al. 2002) but there are no studies comparing the effects of tactile versus proprioceptive stimuli on motor-cortex excitability.

New rehabilitation approaches, such as transcranial magnetic stimulation (TMS) and pharmacological manipulation with antidepressive medication, aim at enhancing plasticity after stroke by altering the excitatory–inhibitory balance of the two hemispheres (Nibuya et al. 1995; Duman et al. 2000; Maya Vetencourt et al. 2008; Chollet et al. 2011; Sung et al. 2013). Despite some promising results, it is still difficult to estimate which patients will benefit from these interventions, in which time window they should be used and what effect these interventions have on the excitability of the two hemispheres. The modulation of the ∽20-Hz motor-cortex rhythm by somatosensory stimulation could be a tool to monitor alterations in motor-cortex excitability for example after stroke and guide the selection of rehabilitation methods.

We examined the ∽20-Hz oscillatory brain activity during tactile stimulation and passive movement of the index fingers in 22 healthy subjects with whole-scalp magnetoencephalography (MEG). The aim was to clarify how these two different somatosensory stimulus types affect motor cortex excitability.

## Materials and Methods

### Subjects

We studied 22 healthy volunteers; 11 males and 11 females, age 42–70 years, mean 59 ± 12 years, all right-handed and with no history of neurological disorders. The local Ethics Committee approved the study protocol and all subjects gave written informed consent prior to the measurements.

### Stimulation

Tactile stimuli (duration 140 ms, peak at 50 ms) were delivered using pneumatic diaphragms driven by compressed air to the tips of both index fingers alternately with an interstimulus interval (ISI) of 1.5 sec, resulting in an ISI of 3 sec for one side.

**Figure 1:**
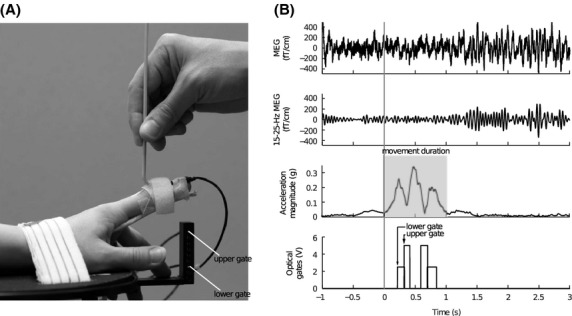
(A) The arrangement for passive movement. (B) Representative signals of one subject during right index finger passive movement. Two upper rows: MEG signal from a single gradiometer channel (raw and filtered 15–25 Hz) over the primary sensorimotor cortex. The ∽20-Hz modulation of the filtered MEG signal is observable even to a single movement. Third row: magnitude of acceleration (i.e*.,* the Euclidean norm of the three accelerations). Total duration of movement is highlighted with gray. Lowest row: trigger signals from the lower (1st) and upper (2nd) optical gates.

For passive movements, an experienced nurse extended briskly the subject's index finger and lowered it back to the initial position, with an ISI of about 3 sec. Right and left index fingers were stimulated separately. To produce as pure proprioceptive stimulation as possible we minimized cutaneous tactile stimulation during the finger lift by covering the middle phalanx with a surgical tape, to which a rigid aluminum stick was attached with a Velcro strap (Fig.1A). This assured that the possible tactile stimulation was as constant as possible. Furthermore, the tip of the finger was not allowed to touch the device. A 3-axis accelerometer (ADXL335 iMEMS accelerometer, Analog Devices Inc., Norwood, MA) was attached on the nail of the index finger and its signals, acquired with the MEG system, were used to determine finger kinematics and the onset of movement (Fig.1).

Two vertically placed optical gates with horizontally placed optical sensors were used to identify successful passive movements. The lower gate was located just above the finger at resting position and the upper gate was located 30 mm higher. The finger was moved through the optical gates, and the passive movement was accepted only if the finger passed through both gates with the lower gate preceding the upper one within 500 ms (Fig.1A). As the movement had already started before the index finger reached the lower gate, the actual onset of the movement was calculated off-line from the accelerometer signals in 17 subjects. In five subjects, the accelerometer signal was not available for technical reasons. These subjects were excluded from the analysis where the information of the exact onset of passive movement was needed (grand average of time-frequency representations; see Data analysis) but retained for other analyzes and statistical tests between the stimulus conditions. For these analyses, the onset of the movement of the five subjects was estimated from the average onset in the other subjects (*N = *17).

The beginning and end of passive movement were determined from the Euclidean norm of the three orthogonal accelerometer channels (i.e., acceleration magnitude), averaged across all accepted passive movements for each subject and each hand separately. The beginning of the movement was set to the transient onset of linear increase in the mean acceleration magnitude signal for each subject separately (Fig.1). The end of the movement was similarly determined as the transient stop of linear decline in the mean acceleration magnitude signal.

### MEG recordings

We employed a 306-channel whole-scalp MEG system (Vectorview™, Elekta Oy, Helsinki, Finland). The helmet-shaped sensor array comprises 102 triple-sensor elements, each housing a magnetometer and two orthogonal planar gradiometers. The data of 18 subjects were recorded in Aalto University (Aalto NeuroImaging, MEG-core) and four with identical measurement and stimulus devices in the BioMag Laboratory (Helsinki University Central Hospital, Helsinki, Finland). During the measurements, the subjects were comfortably seated in a magnetically shielded room in an upright position with the scalp covered by the sensor array. The subjects were instructed to keep their head and posture still, try to avoid excessive blinking, to be relaxed and not to pay attention to the stimuli. Earplugs were used to avoid responses to possible stimulus-related acoustic noise. The nurse was present inside the magnetically shielded room to perform the passive movement of index finger and to observe and guide the subject. The subject's head position with respect to the MEG sensors was determined with the help of four indicator coils (attached to the forehead and mastoids). Prior to the MEG data collection, the locations of the coils and three anatomical landmarks (right and left preauricular points and nasion) as well as 50–100 additional points on the head surface were determined with a 3D digitizer. The head position was measured in each session. Electro-oculogram was utilized to record vertical eye movements. The MEG signals were band-pass filtered to 0.03–330 Hz and digitized at 1000 Hz. Online averaging was performed to monitor the number of accepted trials and continuous data were simultaneously collected for subsequent analysis.

In all recording sessions, tactile stimulation of the right and left index fingers were performed first, followed by a 3-min recording of isometric contraction of both right and left *extensor carpi radialis* for estimating cortex-muscle coherence (the results are reported elsewhere). Thereafter, continuous resting state data were recorded with eyes closed/eyes open for 3 min each. Finally, cortical responses to passive movement of both index fingers were recorded. About 60 accepted trials for each hand were collected for both tactile and passive-movement sessions.

We performed control measurements in four subjects with two different ISIs (1.5 sec and 3 sec) and two durations (140 msec and 1130 msec) of tactile stimuli to test the effects of latency and stimulus duration on the ∽20-Hz rebound.

### Data analysis

To suppress environmental interference, the raw data were first processed with the temporal signal-space separation method (tSSS; Taulu and Simola 2006). Head movement compensation method (Taulu and Kajola 2005; Nenonen et al. 2012) implemented in MaxFilter software (version 2.2.11; Elekta Oy, Helsinki, Finland) was also used.

Amplitude spectra were estimated from the resting state data (eyes open). Half-overlapping 2048-sample Hanning-windowed segments of the continuous data were Fourier-transformed and the magnitudes averaged. For each subject, the MEG channel showing the strongest spectral peak at around 20 Hz over the rolandic region was chosen in both left and right hemispheres. Time–frequency representations (TFR) of both tactile and passive-movement responses in the frequency range of 3–40 Hz were calculated for 17 subjects using 7-cycle Morlet wavelets to determine the frequency band of strongest modulation in the ∽20-Hz range.

Before analyzing tactile stimulus-induced modulations of rhythmic activity, the averaged somatosensory evoked responses were subtracted from each trial of the continuous data. Temporal spectral evolution method (TSE; Salmelin and Hari 1994) was used to quantify the modulation of rhythmic activity; the continuous data were filtered to the frequency band showing the strongest modulation (15–25 Hz in all subjects; determined from the TFR), rectified and averaged time-locked to the stimulus onset. The analysis period was –200–1500 ms for tactile stimulation and –200–2500 ms for passive movement. The 200-ms prestimulus time was used to determine the baseline level for rhythmic activity.

The peak amplitudes of suppression and rebound over the rolandic area were quantified from one channel showing the strongest suppression and another channel showing the strongest rebound of the 15–25-Hz activity both in the ipsi- and contralateral hemisphere to the stimulated hand. The peak latency was determined as the time point where the suppression/rebound was strongest. Thereafter, the relative peak amplitudes were calculated as changes of amplitudes with respect to the individual prestimulus baselines (−200–0 ms) and defined as % values. The relative peak amplitudes and peak latencies of suppressions and rebounds were compared with paired two-tailed *t*-test.

Duration (mean) and acceleration magnitude (mean and peak) were computed to describe kinematics of the passive-movement stimuli. The duration was defined as the time between the beginning and end of passive movement determined from the mean magnitude of the index finger acceleration (see Stimulation). The kinematics between right and left index fingers were compared with paired two-tailed *t*-test.

### Source modeling

Temporal spectral evolution was computed also in the source space in one subject (*S*12) whose head magnetic resonance image (MRI) was available to localize the sources of the strongest rebound. FreeSurfer software (Fischl et al. 1999) was utilized to segment the cranial volume (for a single-compartment boundary element model) and the cortical mantle from a structural 3D head MRI of the subject. Thereafter, cortically constrained L2 minimum norm estimate was computed (“MNE Software”; Gramfort et al. 2014; nlx_151346). Noise covariance was estimated from a 2-min recording without a subject, filtered to 15–25 Hz. MatLab (Mathworks, Natick, MA) functions were written for the TSE calculations in source space.

## Results

### Kinematics of passive movements

Figure1B shows the MEG signal, the acceleration magnitude signal, and the optical gate signal in one subject during a single passive movement of the right index finger. The modulation of the rolandic ∽20-Hz MEG rhythm is discernible.

Kinematics of the passive movement were comparable between the right and left index fingers; no differences were found in the mean (right: 0.19 ± 0.04 g and left: 0.20 ± 0.03 g, *N *=* *17; *p *=* *0.74) and peak (right: 0.40 ± 0.10 g and left: 0.40 ± 0.06 g, *N *=* *17; *p *=* *0.96) acceleration magnitudes or in the mean movement duration (right: 1097 ± 11 ms and left: 1108 ± 10 ms, *N *=* *17; *p *=* *0.30).

### Spontaneous brain activity

At rest with eyes open, amplitude spectra around 20 Hz showed typically 2–4 peaks over the rolandic region at 15–28 Hz in the left and 15–25 Hz in the right hemisphere.

### Modulation of the ∽20-Hz rhythm

**Figure 2:**
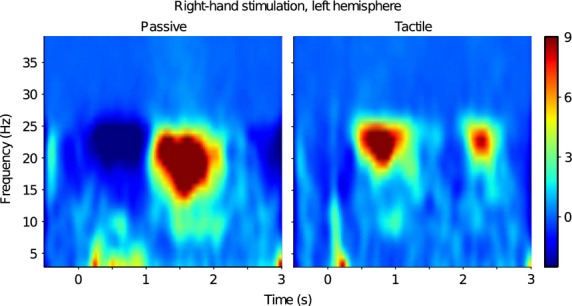
Induced responses to passive movement and tactile stimulation. Grand average (*N *=* *17) time–frequency representations (TFR) to passive movement and tactile stimulation of right index finger. The maximum modulation occurs in the range of 15–25 Hz.

**Figure 3:**
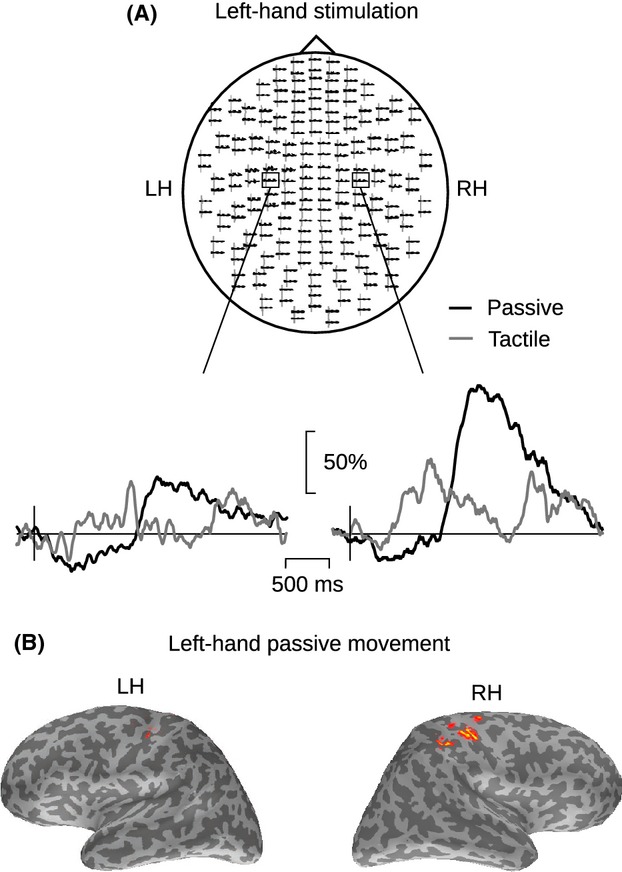
Temporal–spectral evolution (TSE) of the ∽20-Hz rhythm in one subject. (A) Sensor-level TSE of the 15–25-Hz activity to passive movement (black line) and tactile stimulation (gray line) of left index finger in one representative subject (*S*12). The insets show the responses at two planar gradiometer channels. LH, left; RH, right hemisphere. The amplitude scale (vertical) is relative to the baseline level. (B) The cortical source locations (estimated with MNE, see Methods) of the ∽20-Hz modulation in response to left-hand passive movement. The latency of the MNE maps correspond to the strongest rebound, and each cortical surface view was independently thresholded at 60% of its maximum amplitude.

In the TFR analysis, the maximum rebound of the ∽20-Hz rhythm to tactile stimulation and passive movement of the index finger were observed over the same channels as the strongest ∽20-Hz peaks in the amplitude spectra. Figure2 shows the grand average TFR (*N *=* *17). The maximum modulation (suppression and rebound) of ∽20-Hz activity is observed in the 15–25-Hz range in all subjects for both types of stimuli. Therefore, this frequency range was chosen to quantify the modulation of ∽20-Hz activity. Due to the short ISI of 1.5 sec between tactile stimuli of left and right index fingers the subsequent ipsilateral responses are visible (see also Fig.3A).

All subjects showed modulation of ∽20-Hz activity in response to both tactile stimulation and passive movement of the index fingers. Figure3A illustrates ∽20-Hz modulation in one subject (*S*12). Modulation of the rhythm was observed bilaterally to unilateral stimulation but it was stronger in the hemisphere contralateral to the stimulated hand. Figure3B illustrates source locations of the maximum rebound in one subject. The strongest source is located in the anterior part of the contralateral central sulcus.

Maximum rebound and suppression amplitudes were not observed over the same channel in most subjects. The maximum rebound was found in a more anterior channel than the maximum suppression in 20/22 subjects for tactile stimulation and in 19/22 for passive movement, suggesting different generator areas for these two components.

**Figure 4:**
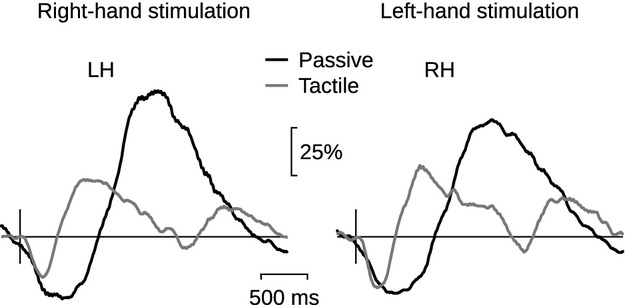
Group-level TSE of the 20-Hz rhythm. Grand average (*N *=* *22) of TSE of the 20-Hz rhythm to passive movement (black line) and to tactile stimulation (gray line) of right and left index finger, in the contralateral left (LH) and right (RH) hemispheres. The amplitude scale (vertical) is relative to the baseline level.

Figure4 shows the grand average of the ∽20-Hz rhythm TSE analysis of 22 subjects. Passive movement elicited a stronger rebound than tactile stimulus. The rebound peak amplitude was on average 35% stronger to passive movement than to tactile stimulation in contralateral hemispheres to right and left index finger stimulation: 95 ± 12% versus 60 ± 8%, (*p < 0.02) to right index finger passive movement and tactile stimulation, respectively, and 89 ± 14% versus 55 ± 6% (**p <* *0.01) to left-sided stimulation.

In contrast, the strength of the suppression did not differ between these two stimulus types: 34 ± 2% versus 28 ± 2%; (*p *=* *0.06) to right index finger passive movement and tactile stimulation, respectively, and 35 ± 2% versus 30 ± 3% (*p *=* *0.13) to left-sided stimulation. The mean peak amplitudes and latencies of the responses to both types of stimuli are listed in Table 1.

**Table 1:** Rebound and suppression amplitudes and peak latencies. The relative amplitudes (mean ± SEM) and peak latencies (mean± SEM) of suppression and rebound to right- and left-hand passive movement and tactile stimulation in both left (LH) and right (RH) hemispheres

	Right-hand stimulation				Left-hand stimulation			
	Passive	Tactile	Passive	Tactile	Passive	Tactile	Passive	Tactile
	LH	LH	RH	RH	RH	RH	LH	LH
Rebound								
Relative amplitude (%)	95 ± 12	60 ± 8	48 ± 6	27 ± 4	89 ± 14	55 ± 6	53 ± 8	28 ± 3
Peak latency (ms)	1420 ± 70	800 ± 50	1420 ± 50	830 ± 50	1440 ± 50	780 ± 40	1430 ± 70	730 ± 50
Suppression								
Relative amplitude (%)	34 ± 2	28 ± 2	31 ± 2	27 ± 2	35 ± 2	30 ± 3	32 ± 2	26 ± 2
Peak latency (ms)	520 ± 20	300 ± 20	590 ± 40	360 ± 20	450 ± 30	270 ± 10	580 ± 40	290 ± 20

**Figure 5:**
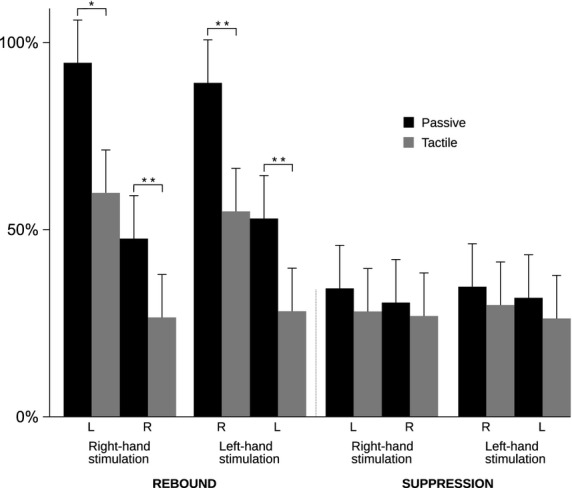
Rebound and suppression magnitudes. Relative (± SEM) strength (%) of the rebound and suppression to right- and left-hand passive movement (black bars) and tactile stimulation (gray bars). *p <* *0.05, **p <* *0.01. LH = left hemisphere, RH = right hemisphere.

Figure5 illustrates the different behavior of rebound and suppression amplitudes in all subjects. The rebound is significantly stronger to passive movement than to tactile stimulation both in the contra- and ipsilateral hemispheres, whereas there is no significant difference between the suppression amplitudes in either hemisphere.

The peak latencies of suppression and rebound did not differ between the hemispheres within either stimulus type. Both suppression and rebound peak latencies were longer to passive movements than to tactile stimuli in both hemispheres (***p <* *0.001) due to different durations of the two stimuli. Durations of suppression and rebound were not compared because of different stimulus lengths.

## Discussion

The present study shows that passive movement is a strong modulator of the ∽20-Hz motor-cortex rhythm. The rebound of the rhythm was significantly stronger to passive movement than to tactile stimulation, whereas the magnitude of the suppression did not differ between these two stimulus types.

A ∽20-Hz rebound is observed after termination of movement or somatosensory stimulation and is therefore associated with deactivation or inhibition of the motor cortex (Salmelin et al. 1995b; Pfurtscheller et al. 1996, 1997; Cassim et al. 2000, 2001). This is further supported by TMS studies showing decreased motor-cortex excitability (decreased motor-evoked potentials) after cutaneous and median-nerve stimulation at latencies comparable with that of the ∽20-Hz rebound (Chen et al. 1999; Abbruzzese et al. 2001). Furthermore, a combined MEG and magnetic resonance spectroscopy study showed a positive correlation between the ∽20-Hz rebound strength and the inhibitory neurotransmitter gamma-aminobutyric acid (GABA) concentration, suggesting that the rebound represents a period of GABAergic inhibition in MI (Gaetz et al. 2011).

Afferent somatosensory input has been proposed to affect motor functions by modulating motor-cortex excitability (Asanuma et al. 1979; Asanuma and Arissian 1984; Favorov et al. 1988; Ridding and Rothwell 1999a; Cassim et al. 2001; Houdayer et al. 2006; Reyns et al. 2008). In accordance, tactile or median-nerve stimulation alone, with no active movement, is sufficient to elicit a ∽20-Hz rebound, reflecting the modulatory effect of afferent input on motor-cortex activity (Salmelin and Hari 1994; Schnitzler et al. 1995, 1997; Pfurtscheller and Neuper 1997; Salenius et al. 1997; Hari et al. 1998, 2014; Neuper and Pfurtscheller 2001; Stancák et al. 2003; Pfurtscheller et al. 2005; Laaksonen et al. 2012). The rebound is abolished by blocking afferent input under ischemia (Cassim et al. 2001) or by sensory deafferentation (Reyns et al. 2008). Moreover, Cassim et al. (2000) showed prolonged synchronization of the ∽20-Hz rhythm during sustained isometric contraction, suggesting that the rebound is related to continuous flow of afferent proprioceptive input. Hence, the increase in the ∽20-Hz rhythm might represent ongoing coordination of sensory input and motor output (Gaetz and Cheyne 2006). These studies suggest that afferent input—but not movement as such—is necessary and sufficient to elicit the ∽20-Hz rebound.

Animal studies have shown that the primary motor cortex (MI) receives proprioceptive input via direct thalamocortical connections (Asanuma et al. 1979), via primary somatosensory cortex (SI; areas 3a and 2) and via secondary somatosensory cortex (SII) (Jones and Wise 1977; Jones et al. 1978; Jones 1983). Studies in monkeys have shown that the connections from area 3a (the primary projection area for proprioception) to MI are faster and more direct than from area 3b (primary projection area for tactile input), which has no or only sparse connections to MI (Jones et al. 1978; Jones 1983; Mima et al. 1997). In line with that, input from proprioceptors arrives to MI at similar short latencies (5–10 ms) as to SI (Devanandan and Heath 1975; Lucier et al. 1975).

In accordance, the present study showed that the rebound was stronger to passive movement than to tactile stimulation. This is congruent with earlier studies showing that magnitude of rebound might depend on activated musvle mass (Pfurtscheller et al. 1998) and voluntary movement and mixed-nerve stimulation elicit a stronger rebound than pure tactile stimulation (Houdayer et al. 2006). Furthermore, selective laser stimulation of nociceptive C-fibers did not produce any rebound, whereas a weak rebound was observed after stimulation of A*δ* fibers (Raij et al. 2004). These studies suggest that the magnitude of the ∽20-Hz rebound depends on the type and quantity of afferent input. Tactile stimulation activates mainly exteroceptive afferents, whereas passive movement activates primarily proprioceptors and to a lesser extent exteroceptors. The stronger rebound to passive movement indicates stronger interaction of proprioceptive versus tactile input with motor output.

There are substantial differences in the proprioceptive and tactile stimulation in the present study that may have had an effect on rebound. Stimulus duration in passive movement (1130 msec) is significantly longer than in tactile stimulation (140 msec). However, as the tactile stimulus activates rapidly adaptive cutaneous receptors, it is not likely that stimulus duration would significantly affect the rebound peak amplitude although it might have an effect on rebound duration. Accordingly, our control measurements with tactile stimuli presented with durations of 140 msec and 1130 msec showed no difference in rebound peak amplitude although it prolonged the rebound duration.

Tactile stimuli were delivered to index fingers alternately with an interstimulus interval (ISI) of 1.5 sec, resulting in an ISI of 3 sec for one side, whereas passive movements were performed with a 3 sec ISI for one side at the time. As unilateral stimulation has a bilateral effect on motor-cortex oscillations, although much weaker in ipsilateral side, the tactile stimuli occurring at 1.5 sec to the ipsilateral side could have an effect on the rebound. However, the rebound peaked to tactile stimuli at 793 ± 34 msec, that is much earlier than the subsequent ipsilateral stimulus arriving at 1500 msec, and therefore the ipsilateral stimulus is not likely to affect the peak amplitude. Furthermore, an earlier study using the same tactile stimulus in healthy subjects with an ISI of 3005 msec (Laaksonen et al. 2012) showed similar rebound amplitudes (57 ± 5% vs. 61 ± 11%) than the present study with an alternating 1.5 sec ISI, indicating that the shorter ISI does not affect the peak amplitude nor peak latency of the rebound.

The suppression of the ∽20-Hz rhythm starts within 500 msec after movement or somatosensory stimulation onset and is believed to reflect increased activity of the motor cortex (Salmelin et al. 1995a; Hari et al. 1997, 1998; Pfurtscheller and Lopes da Silva 1999; Müller et al. 2003; Raij et al. 2004). Similarly, suppression of this rhythm has been observed to painful laser stimuli at latencies comparable with the estimated conduction velocities of the two nociceptive fiber systems, which suggests that also noxious input excites (or disinhibits) MI. In addition, reaction times for lifting the index finger to tones were observed to be shorter during suppression induced by noxious input than before the onset of the 20-Hz suppression, indicating facilitation of the motor cortex (Raij et al. 2004). Even motor imagery causes suppression in the ∽20-Hz rhythm (Hari et al. 1998), and the suppression has been associated with significantly higher motor-evoked potentials, reflecting increased MI activation (Takemi et al. 2013).

The suppression and rebound of the ∽20-Hz rhythm have been reported to be generated within the same cortical region and have been interpreted to represent different levels of activation of the same neuronal population (Pfurtscheller 1992; Szurhaj et al. 2003). In the present study, we found that the stimulus type affected the suppression and rebound differently. This finding is in line with studies showing no difference in the suppression magnitude while the rebound amplitude varied between rapid and slow finger movements (Stancák and Pfurtscheller 1995, 1996), index and four-finger flexion (Salmelin et al. 1995a), brief ballistic wrist movement and onset of sustained isometric wrist extension (Alegre et al. 2002) and different types of ballistic movements (Wheaton et al. 2009). Furthermore, the peak frequencies of suppression (∽21 Hz) and rebound (∽19 Hz) have been shown to differ slightly (Pihko et al. 2014), and suppression and rebound have even occurred simultaneously in different beta bands (Pfurtscheller et al. 1997). The location of the maximum ∽20-Hz suppression typically appears posterior to the central sulcus, in the vicinity of the SI hand area, while the rebound is often localized anterior to the central sulcus in the MI hand region (Salmelin et al. 1995a,b; Pfurtscheller et al. 1996; Jurkiewicz et al. 2006). Accordingly, in our study the strongest rebound was observed over more anterior MEG channels than the maximal suppression.

Taken together, there is accumulating evidence that the neuronal populations generating ∽20-Hz suppression and rebound are both anatomically and functionally distinct. The suppression seems to be more independent of the type and quantity of somatosensory input and might thus reflect “all or none” -type of activation of the motor cortex, whereas the rebound is modulated strongly according to the type and strength of somatosensory input.

The peak latency of the ∽20-Hz rebound to both tactile and proprioceptive stimulation is long (in the present study 680–1500 ms). In addition to direct thalamocortical connections, MI receives afferent input from SI, SII, posterior parietal cortex and supplementary motor area that are activated for several hundreds of milliseconds after a somatosensory stimulus (Jones and Wise 1977; Jones et al. 1978; Shibasaki et al. 1980; Jones 1983; Mori et al. 1989; Donoghue and Sanes 1994; Hari et al. 1990; Weiller et al. 1996; Alary et al. 1998, 2002; Disbrow et al. 2000; Druschky et al. 2003). It is possible that the rebound reflects inflow of afferent input from these multiple higher order somatosensory areas. Furthermore, a weak positive correlation has been found between the rebound strength and the amplitude of SII evoked responses in stroke patients, whereas the SI response amplitudes did not correlate with the rebound (Laaksonen et al. 2012). Interaction of SII and motor cortex is demonstrated also by some pathological conditions such as Unverricht–Lundborg disease, Parkinson's disease and focal dystonia where deficient SII activation and altered motor-cortex excitability have been observed in the same patients (Boecker et al. 1999; Silén et al. 2000; Abbruzzese et al. 2001; Forss et al. 2001).

A previous study (Laaksonen et al. 2012) indicated that the ∽20-Hz rebound strength is correlated with the recovery of hand function after stroke. The present results show that proprioceptive stimulation elicits an even stronger rebound of the ∽20-Hz rhythm than tactile stimulation. Hence, modulation of the ∽20-Hz rhythm to passive movement, which does not require an active contribution of the subject and is not affected by changes in tactile sensitivity, could be a useful tool to evaluate alterations in cortical excitability after stroke. As the arising possibilities to boost recovery from stroke aim at modifying the excitatory–inhibitory balance of the brain, their effectiveness could be objectively evaluated by monitoring alterations in the ∽20-Hz rebound.

## Conclusions

In the present study, passive movement elicited a stronger ∽20-Hz rebound than tactile stimulation, indicating that proprioceptive input modulates motor-cortex excitability more than tactile input. However, the magnitude of suppression did not differ between these two different stimuli, suggesting that suppression and rebound may represent activity of two functionally distinct neuronal populations**.**
